# Butyrylcholinesterase activity in patients with postoperative delirium after cardiothoracic surgery or percutaneous valve replacement- an observational interdisciplinary cohort study

**DOI:** 10.1186/s12883-024-03580-9

**Published:** 2024-03-01

**Authors:** Konstantin Schlake, Johannes Teller, Lukas Hinken, Hans Laser, Ralf Lichtinghagen, Andreas Schäfer, Christine Fegbeutel, Karin Weissenborn, Carolin Jung, Hans Worthmann, Maria Magdalena Gabriel

**Affiliations:** 1 Department of Neurology, Hannover Medical School, Carl-Neuberg-Str. 1, 30625 Hannover, Germany; 2 Department of Anaesthesiology and Intensive Care Medicine, Hannover Medical School, Carl-Neuberg-Str. 1, 30625 Hannover, Germany; 3 Department for Educational and Scientific IT Systems, Hannover Medical School, MHH Information Technology, Carl-Neuberg-Str. 1, 30625 Hannover, Germany; 4 Institute of Clinical Chemistry, Hannover Medical School, Carl-Neuberg-Str. 1, 30625 Hannover, Germany; 5 Cardiac Arrest Center, Department of Cardiology and Angiology, Hannover Medical School, Carl-Neuberg-Str. 1, 30625 Hannover, Germany; 6 Department of Cardiothoracic, Transplantation and Vascular Surgery, Hannover Medical School, Carl-Neuberg-Str. 1, 30625 Hannover, Germany

**Keywords:** Butyrylcholinesterase, Postoperative delirium, Cardiac surgery, Percutaneous valve replacement, Risk factors

## Abstract

**Background and objectives:**

Postoperative delirium is a frequent and severe complication after cardiac surgery. Activity of butyrylcholinesterase (BChE) has been discussed controversially regarding a possible role in its development. This study aimed to investigate the relevance of BChE activity as a biomarker for postoperative delirium after cardiac surgery or percutaneous valve replacement.

**Methods:**

A total of 237 patients who received elective cardiothoracic surgery or percutaneous valve replacement at a tertiary care centre were admitted preoperatively. These patients were tested with the Montreal Cognitive Assessment investigating cognitive deficits, and assessed for postoperative delirium twice daily for three days via the 3D-CAM or the CAM-ICU, depending on their level of consciousness. BChE activity was measured at three defined time points before and after surgery.

**Results:**

Postoperative delirium occurred in 39.7% of patients (*n* = 94). Univariate analysis showed an association of pre- and postoperative BChE activity with its occurrence (*p* = 0.037, *p* = 0.001). There was no association of postoperative delirium and the decline in BChE activity (pre- to postoperative, *p* = 0.327). Multivariable analysis including either preoperative or postoperative BChE activity as well as age, MoCA, type 2 diabetes mellitus, coronary heart disease, type of surgery and intraoperative administration of red-cell concentrates was performed. Neither preoperative nor postoperative BChE activity was independently associated with the occurrence of postoperative delirium (*p* = 0.086, *p* = 0.484). Preoperative BChE activity was lower in older patients (B = -12.38 (95% CI: -21.94 to -2.83), *p* = 0.011), and in those with a history of stroke (B = -516.173 (95% CI: -893.927 to -138.420), *p* = 0.008) or alcohol abuse (B = -451.47 (95% CI: -868.38 to -34.55), *p* = 0.034). Lower postoperative BChE activity was independently associated with longer procedures (B = -461.90 (95% CI: -166.34 to -757.46), *p* = 0.002), use of cardiopulmonary bypass (B = -262.04 (95% CI: -485.68 to -38.39), *p* = 0.022), the number of administered red cell-concentrates (B = -40.99 (95% CI: -67.86 to -14.12), *p* = 0.003) and older age (B = -9.35 (95% CI: -16.04 to -2.66), *p* = 0.006).

**Conclusion:**

BChE activity is not independently associated with the occurrence of postoperative delirium. Preoperative BChE values are related to patients’ morbidity and vulnerability, while postoperative activities reflect the severity, length and complications of surgery.

**Supplementary Information:**

The online version contains supplementary material available at 10.1186/s12883-024-03580-9.

## Introduction

The American Geriatrics Society recognises postoperative delirium (POD) as the most frequent complication after surgery in elderly patients [1]. It is associated with longer hospital length of stay, higher mortality rates and cognitive and functional decline even months after.

discharge [2–4].

Reported incidences vary between 4 and 55%, with a dramatic increase in mechanically ventilated patients [5, 6].

Identifying patients at risk for POD could combat underdiagnoses, improve patient surveillance and lead to early treatment, which is critical in improving patients’ outcomes. In past studies, an association between POD and the dynamics of BChE and AChE activity—the two main ACh cleaving enzymes—has been demonstrated in surgical patients [7].

A cholinergic deficit, reflected by an imbalance of acetylcholinesterase (AChE) and butyrylcholinesterase (BChE) activity, may lead to a disrupted synaptic transmission and thus impair cognitive function. Particularly in older patients with invasive cardiac procedures but also with transcatheter aortic valve implantation (TAVI), the assessment of BChE activity and its decline has been evaluated as a biomarker for POD with inconsistent results [8].

Different suggestions have been made regarding the timing (pre- vs. postoperative) and method (total value vs. delta) of BChE measurement as a predictor for POD [9, 10].

Clinical trials addressing the use of physostigmine for treatment of the hypothesized cholinergic deficit yielded controversial results, recently with failure in preventing POD [11].

Different explanations for the pathophysiology of POD are discussed. The neurotransmitter hypothesis focuses on the imbalance between acetylcholine, dopamine and serotonin in the central nervous system, postulating an acetylcholine deficiency combined with a dopamine or norepinephrine excess and – depending on the type of delirium – a serotonin excess or deficiency [12, 13].

In contrast, the neuroinflammatory hypothesis suggests a dysregulation of inflammatory and anti-inflammatory pathways, resulting in an exaggerated response to peripheral inflammatory stimuli such as surgical trauma. The proinflammatory reaction is characterised by a release of cytokines like TNF-α. By crossing the blood–brain-barrier they bind to microglial cells, which in turn produce mediators that impair the cholinergic -metabolism and alter neurotransmission causing delirious symptoms [12, 14, 15].

With these two different hypotheses in mind, we investigated the course of the BChE activity in patients undergoing cardiac surgery or percutaneous valve replacement.

## Methods

This study is an observational interdisciplinary cohort study of delirium as a neurological complication after cardiac surgery and percutaneous valve replacement. Recently, a study focusing on the neurological complications from this cohort has been published [16].

The study was performed at an academic tertiary care centre, including patients undergoing elective cardiothoracic surgery or cardiac valve replacement between August 2018 and March 2019. Due to a lack of follow-up blood samples in some patients the cohort described in this paper is slightly different from the one of the previous report [16].

**Fig. 1:**
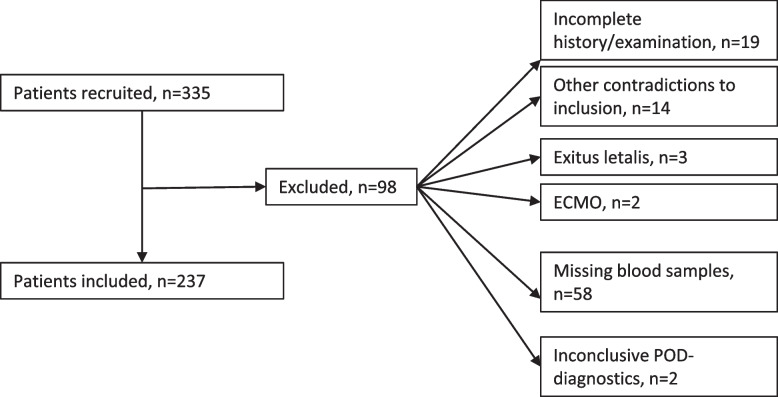
Flow-chart of patient inclusion and exclusion

Exclusion criteria for the current analysis were acute infection (diagnosed via inflammation markers and clinical presentation), emergency or revisional surgery, malignoma or chemotherapy within the last two years, dementia, known immunodeficiency and recent alcohol or drug abuse as well as focal neurological deficits prior to surgery intervention (Fig. 1).

A neurological examination, a neurocognitive assessment using the Montreal-Cognitive-Assessment-Test (MoCA), the malnutrition screening tool (MST) and the patient health questionnaire 2 (PHQ-2) were performed the day before intervention and categorized as described previously [17–19].

Medical history was collected. Intraoperative data such as length of surgery, medication, red cell concentrate (RCC)/fresh frozen plasma (FFP) transfusion and the use and duration of cardiopulmonary bypass (CPB) were recorded.

Patients were screened for the occurrence of POD twice daily on the first, second and third postoperative day with either the Confusion Assessment Method (3D-CAM) or the Confusion Assessment Method Intensive Care Unit (CAM-ICU), depending on the Richmond Agitation-Sedation Scale (RASS); RASS -1 to + 1 resulted in 3D-CAM and otherwise CAM-ICU was used [20–22]. All of these tests are well-established methods and have been used commonly in international studies previously.

Categorization of the delirium-subtype was carried out via RASS-score (hypoactive = below zero, hyperactive = above zero, mixed = above and below zero within screening periods). In addition to routinely taken blood samples, plasma samples were obtained pre- and postoperatively (day 3), processed and promptly frozen at -80 C.

An additional blood sample was taken if the patient was diagnosed with POD. Routine blood parameters included bilirubin, C-reactive protein, lactate dehydrogenase (LDH), leucocytes, glucose, hemoglobin, potassium, sodium, calcium, lactate and creatinine. Clinical data were extracted from the hospital documentation system by the Enterprise Clinical Research Data Warehouse (ECRDW) of the MHH Information Technology. BChE activity was analysed via photometric measurement (LISA-CHE®-POC-device, Dr. Franz Köhler Chemie GmbH, Bensheim, Germany) according to the producer’s instructions. 

All patients gave written informed consent. The study was approved by the local ethics committee on the 06.06.2018 (Ethics Committee of Hannover Medical School, Hannover, Germany, Approval No. 7876 BO S 2018 by Univ. Prof. Dr. med. Stefan Engeli) and conducted in accordance with their relevant guidelines and regulations. The study was conducted in accordance with the Declaration of Helsinki. It was conducted according to the guidelines of "The Strengthening the Reporting of Observational Studies in Epidemiology” (STROBE).

### Statistics

Statistical analysis was performed using SPSS © Statistics, Version 28 (©1989, 2021 by SPSS Inc., Chicago, Illinois, USA). Results are displayed as mean ± standard deviation SD, median (25th-75th percentile) or frequencies (percentages). Univariate associations were tested with the Pearson χ^2^-test, Pearson’s R or Spearman’s Rho test, depending on data distribution. Univariate analysis was performed using Mann–Whitney-U-test or Student’s t-test depending on data distribution. A receiver operating characteristic (ROC) analysis including Youden’s Index was used to test the predictive quality of preoperative BChE for the development of POD.

For all multivariable analyses, only parameters that had a significant univariate association with the independent variable were considered. Due to the high number of potential confounders, the final choice of variables that were entered into the multivariable models was discussed in an interdisciplinary setting with the coauthors to avoid the use of multiple confounders or over-correction for confounding. For the analysis of POD, the preoperative and postoperative BChE activity was analyzed in two different models based on a definite interdependence of these parameters. The first POD model included only the preoperative BChE activity and other potentially predictive parameters (supplementary Table 2). In the second POD model, postoperative BChE activity as well as additional intra- and postoperative variables (supplementary Table 3) were analyzed. In the following, variables influencing preoperative and postoperative BChE activity were investigated. Accordingly, for the influence on preoperative BChE activity, only preoperative influencing factors were taken into account. To examine the influencing variables on the postoperative BCHE activity, pre-, peri-, and postoperative parameters were considered. A backwards stepwise multivariate linear regression or a logistic regression model was used to identify independent confounding factors. P-values of less than 0.05 were considered statistically significant.

## Results

### Postoperative delirium

A total of 335 patients were screened and 237 patients were finally included after consideration of the in- and exclusion criteria. All in all, 98 patients had to be excluded due to: Missing blood samples (*n* = 58), incomplete medical history or examination (*n* = 19), inconclusive POD-diagnostics (*n* = 2), exitus letalis (*n* = 3), ECMO (*n* = 2) or other criteria (no surgery (*n* = 3), recent malignoma (*n* = 2), endocarditis (*n* = 2), emergency or revisional surgery (*n* = 2), recent abusive alcohol use (*n* = 1), preoperative delirium (*n* = 1), dementia (*n* = 1), pituitary adenoma (*n* = 1), HIV (*n* = 1) (Fig. 1).

Baseline characteristics, including the distribution of surgical procedures, are given in Table 1 (Table 1).

**Table 1:** Baseline clinical characteristics of all patients and those with and without postoperative delirium

	**Total (*n* = 237)**	**POD (*n* = 94)**	**No POD (*n* = 143)**	***P*- value**
Female Sex	89 (37.6%)	36 (38.3%)	53 (37.1%)	.848
Age (years)	74 (64–79.5)	76 (70–81)	71 (62–79)	**.001**
BMI (kg m^−2^)	26.9 (24.1–30.1)	26.6 (24–29.8)	27.1 (24.2–30.1)	.541
Type 2 diabetes mellitus	65 (27.4%)	33 (35.1%)	32 (22.4%)	**.032**
Coronary heart disease	206 (86.9%)	89 (94.7%)	117 (81.8%)	**.004**
Heart failure	130 (54.9%)	59 (62.8%)	71 (49.7%)	**.047**
NYHA				**.011**
0-I	115 (48.5%)	36 (38.3%)	79 (55.2%)	
II-IV	122 (51.5%)	58 (61.7%)	64 (44.8%)	
Arterial hypertension	51 (21.5%)	24 (25.5%)	27 (18.9%)	.223
History of myocardial Infarction	35 (14.8%)	18 (19.1%)	17 (11.9%)	.123
History of atrial fibrillation	67 (28.3%)	24 (25.6%)	43 (30.0%)	.448
History of chronic alcoholism	19 (8.0%)	7 (7.4%)	12 (8.4%)	.793
History of stroke	24 (10.1%)	13 (13.8%)	11 (7.7%)	.125
History of delirium	15 (6.3%)	9 (9.6%)	6 (4.2%)	.096
History of surgery < 6 months	18 (7.6%)	10 (10.6%)	8 (5.6%)	.152
History of depression	18 (7.6%)	6 (6.4%)	12 (8.4%)	.569
History of malignoma	29 (12.2%)	17 (18.1%)	12 (8.4%)	**.026**
MoCA-score preoperative	25 (22–26)	23 (21–26)	25 (23–27)	** < .001**
PHQ-2				.322
0	155 (65.4%)	59 (62.8%)	96 (67.1%)	
1	36 (15.2%)	13 (13.8%)	23 (16.1%)	
2	27 (11.4%)	12 (12.8%)	15 (10.5%)	
3	8 (3.4%)	2 (2.1%)	6 (4.2%)	
4	11 (4.6%)	8 (8.5%)	3 (2.1%)	
MST ≥ 3 points	11 (4.6%)	5 (5.3%)	6 (4.2%)	.688
BChE activity preoperative (U l^−1^)	2741.1 (2139.7–3286.9)	2558.5 (2007.7–3148.7)	2867 (2249.2–3432.7)	**.044**
TAVI/MitraClip	41 (17.3%)	11 (11.7%)	30 (21%)	.065
Valve-surgery + CPB	58 (24.5%)	18 (19.1%)	40 (42.6%)	.122
Bypass-surgery + CPB	76 (32.1%)	28 (29.8%)	48 (33.6%)	.542
Bypass-surgery (without CPB)	17 (7.1%)	10 (10.6%)	7 (4.9%)	.094
Vascular-aortic surgery	6 (2.5%)	5 (5.3%)	1 (0.7%)	.027
Valve + bypass-surgery	35 (14.8%)	20 (21.3%)	15 (10.5%)	.022
Transapical valve-surgery	4 (1.7%)	2 (2.1%)	2 (1.4%)	.670

Data are presented as median (25 th-75th percentile) or proportions. Baseline parameters were collected and the groups were compared using *χ*^*2*^-tests or *Mann–Whitney-U*, respectively. Statistically significant results are shown in **bold**, *p* < .05 was considered significant

*BMI* Body Mass Index, *NYHA* New York Heart Association, *MoCA* Montreal Cognitive Assessment, *PHQ-2* Patient Health Questionnaire-2, *MST* Malnutrition Screening Tool, *BChE* Butyrlycholinesterase, *TAVI* Transcatheter Aortic Valve Implantation, *CPB* Cardiopulmonary Bypass

All in all, 94 patients (39.7%) developed POD. Of these 94 patients, 73 (77.7%) were screened positive on the first, 14 (14.9%) on the second and 7 (7.4%) on the third postoperative day. Hyperactive delirium was the most common form with 50 patients (53.2%), followed by mixed POD with 32 patients (34.0%) and lastly hypoactive delirium with 12 patients (12.8%).

In univariate analysis, patients with POD were older and suffered more frequently from comorbidities and their preoperative BChE levels were lower (*p* = 0.044) (Table 1).

Further intra- and postoperative parameters are shown in Table 2 (Table 2).

**Table 2:** Peri- and postoperative variables for patients with and without POD

	**POD (*n* = 94 pat.)**	**No POD (*n* = 143 pat.)**	***P*- value**
**Perioperative variables**			
Length of surgery (min)	196 (160–246)	182 (128–225)	**.028**
Type of anaesthesia			.068
General anaesthetic	85 (90.4%)	117 (81.8%)	
Analgosedation	9 (9.6%)	26 (18.2%)	
Use of CPB	69 (73.4%)	99 (69.2%)	.489
Time on CPB (min)	109 (83–137)	102 (80–134)	**.**355
Cross-clamped aorta	68 (72.3%)	96 (67.1%)	.396
Cross-clamp-time (min)	65 (44–79)	60 (46–80)	.435
MAP (mean)	67 ± 5	68 ± 5	.318
RCCs intraoperatively	38 (40.4%)	26 (18.2%)	** < .001**
RCCs total	75 (79.8%)	66 (46.2%)	** < .001**
FFPs intraoperatively	6 (6.4%)	5 (3.5%)	.281
FFPs total	21 (22.3%)	16 (11.2%)	**.018**
**Postoperative variables**			
CRP (mg l^−1^)	174.4 (125.2–243.8)	160.9 (84–211.5)	**.022**
Leukocytes (^*^1000 µl^−1^)	13.5 (10.3–17.5)	11.8 (9.7–15.3)	.072
Lactate (mmol l^−1^)	2.6 (1.9–4.0)	2.1 (1.7–2.6)	** < .001**
Haemoglobine (g dl^−1^)	8.3 (7.6–8.8)	8.8 (8.2–9.6)	** < .001**
Length of stay (d)	9 (7–12)	8 (7–10)	**.008**
Postoperative BChE activity (U l^−1^)	1463.8 (1217.6–1757.8)	1715.1 (1327.1–2120.8)	**.001**
Decline of BChE activity (pre- to postoperative) (%)	39.75 (23.93–53.68)	38.80 (19.60–52)	.327

Data are presented as median (25th-75th percentile) or proportions. Intra- and postoperative parameters were collected and the groups were compared using χ2-tests or Mann–Whitney-U test, respectively. Statistically significant results are shown in **bold**, *p* < .05 was considered significant

*POD* Postoperative Delirium, *CPB* Cardiopulmonary bypass, *MAP* Mean arterial pressure, *RCC* Red-cell concentrate, *FFP* Fresh frozen plasma, *CRP* C-reactive Protein, *BChE* Butyrylcholinesterase

In view of its univariate association, the predictive value of preoperative BChE activity for the development of POD was investigated with a ROC-analysis. An activity of 2379.7 U/ml was calculated as the optimal cut-off-point via Youden-Index and resulted in a sensitivity of 42.6% and a specificity of 72.7% regarding the prediction of POD.

In a binomial logistic regression model including the preoperative BChE activity, age, preoperative MoCA-score, history of coronary heart disease and type 2 diabetes mellitus, significantly higher odds for the development of POD could be found for patients with coronary heart disease (OR = 4.371 (95% CI: 1.502 to 12.721), *p* = 0.007). Patients with a higher preoperative MoCA-score had lower odds of developing POD (OR = 0.823 (95%CI: 0.742 to 0.913).

There was no significant independent association of POD with the preoperative BChE activity (OR = 0.971 (95% CI: 0.939 to 1.004), *p* = 0.086), age (OR = 1.027 (95%CI: 0.998 to 1.057), *p* = 0.065) or type 2 diabetes mellitus (OR = 1.508 (95%CI: 0.800 to 2.842), *p* = 0.204).

In a second binomial regression model, the association of POD with postoperative BChE activity was investigated. In addition to the first model the type of surgery (TAVI/MitraClip or other surgeries) and the intraoperative administration of RCCs (dichotomous variable) were included. Significantly higher odds for developing POD were found for patients with older age (OR = 1.058 (95% CI: 1.020 to 1.098), *p* = 0.002), coronary heart disease (OR = 4.491 (95% CI: 1.463 to 13.783), *p* = 0.009) and intraoperative administration of RCCs (OR = 2.033 (95%CI: 1.005 to 4.113), *p* = 0.048). Patients with a higher MoCA (OR = 0.823 (95% CI: 0.741 to 0.915), *p* < 0.001) and less invasive procedures such as TAVI/MitraClip (OR = 0.222 (95%CI: 0.077 to 0.640), *p* = 0.005) were less likely to develop delirium. There was no association for postoperative BChE activity (OR = 0.981 (95%CI: 0.929 to 1.036), *p* = 0.484) or type 2 diabetes mellitus (OR = 1.631 (95%CI: 0.831 to 3.199), *p* = 0.155), respectively. Regression coefficients and standard deviations in addition to the results mentioned in the text can be found in the supplementary material. The delta in BChE activity pre- to postoperatively was not associated with the development of POD (*p* = 0.327) in univariate analysis.

### Preoperative BChE values

Clinical and epidemiological variables were tested for their univariate association with preoperative BChE activity and are provided in the supplement.

Lower preoperative levels of BChE were not associated with preexisting conditions such as coronary heart disease (*p* = 0.428), type 2 diabetes mellitus (*p* = 0.251) or POD in patient history (*p* = 0.176). Instead, we found an association with age (*p* = 0.003), a history of alcohol abuse (*p* = 0.028) and history of stroke (*p* = 0.003).

These findings could be verified in a linear regression model that indicated an independent association of preoperative BChE activity with age (B = -12.383 (95% CI: -21.937 to -2.829), *p* = 0.011), history of stroke (B = -516.173 (95% CI: -893.927 to -138.420), *p* = 0.008) and a positive history of alcohol abuse (B = -451.466 (95% CI: -868.380 to -34.551), *p* = 0.034).

### Postoperative BChE values

The influence of various pre-, intra- and postoperative variables was tested with regard to the impact on the postoperative BChE level at day 3 and are provided in the supplement.

Values reflecting preclinical and clinical patient condition as well as the severity of surgery, represented by inflammation levels, lactate, length of surgery and transfusions required were considered. Included in the regression model were the following variables: Age, BMI, type 2 diabetes mellitus, history of stroke, type of surgery, total number of administered RCCs, and intraoperative lactate concentrations. In a multivariable regression model age (B = -9.353 (95% CI: -16.043 to -2.664), *p* = 0.006), use of CPB (B = -262.038 (95% CI: -485.684 to -38.393), *p* = 0.022) and the total number of RCC-transfusions (B = -40.991 (95% CI: -67.864 to -14.117), *p* = 0.003) were independently associated with a lower postoperative BChE activity. The median (25th to 75th percentile) number of administered RCCs in total was 3 (2–6) in patients with POD and 0 (0–3) in those without.

A higher BMI (B = 24.231 (95% CI: 11.069 to 37.393), *p* < 0.001) and less invasive surgeries such as TAVI and MitraClip (B = 461.902 (95% CI: 166.339 to 757.464), *p* = 0.002), were independently associated with a higher postoperative BChE activity. There was no independent association of postoperative BChE activity and a history of stroke (B = -204.250 (95% CI: -438.515 to 30.016), *p* = 0.087), type 2 diabetes mellitus (B = 112.241 (95%CI = -53.025 to 277.507), *p* = 0.182), nor with intraoperative lactate values (B = -1.929 (95%CI -61.570 to 57.712), *p* = 0.949).

### Decline in BChE activity

**Fig. 2:**
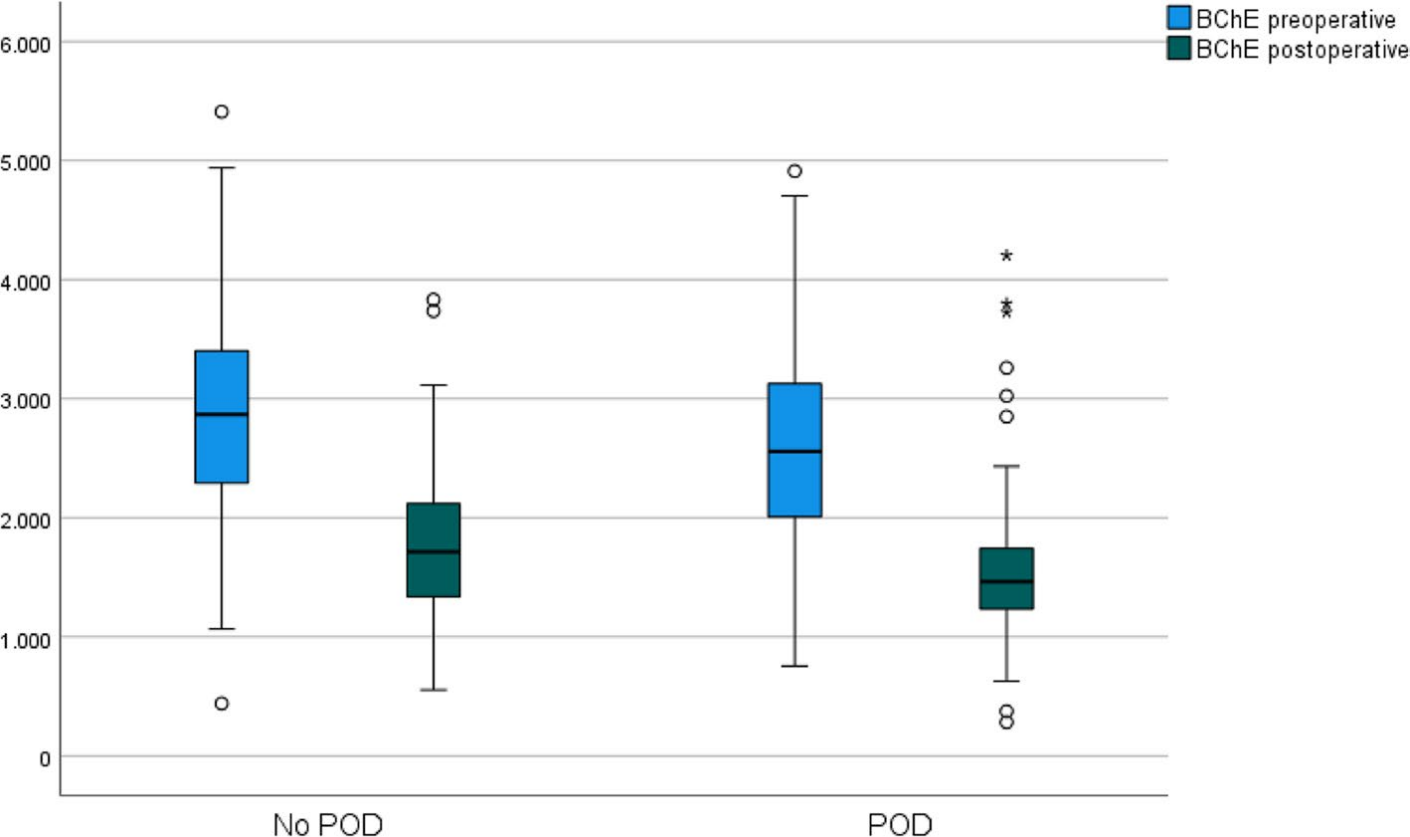
Boxplot of Butyrylcholinesterase-activities (BChE) (U l^−1^) in patients with and without Postoperative Delirium (POD). Preoperative (*blue*) and postoperative (*green*) BChE activities (U l^−1^) for patients with and without postoperative delirium

The dynamic of BChE activity during the observation period is shown in Fig. 2 (Fig. 2), influence of pre-, intra- and postoperative variables are provided in the supplement. Apart from univariate association with age (*p* = 0.037) arterial hypertension (*p* = 0.045) and female sex (*p* = 0.040), analysis of BChE decline did not provide any substantial new findings compared with postoperative activities.

## Discussion

In this study the association between POD and the pre- and postoperative BChE activity was examined in patients with cardiothoracic surgery or percutaneous valve replacement.

The main findings of this study are that (i) although pre- and postoperative BChE activity decreased in patients with POD compared to those without, we could not find any independent association between POD development and the BChE activity. (ii) Instead, BChE activity serves as a marker of chronic and acute inflammation. It was significantly lower in older patients, those who received more invasive surgeries as well as RCCs, and those on cardiopulmonary bypass.

The role of BChE activity in the context of POD-development has been addressed in the past with the intention of simplifying the clinical routine by identifying high-risk patients or early stages of POD. Inconsistent results in previous studies, heterogeneous outcome parameters, and small study groups prompted us to conduct a profound investigation of the role of BChE activity considering various clinical parameters that are known possible risk factors for the development of POD. A predictive biomarker would be of great value in this population of elderly patients highly prone to postoperative complications. Although pre- and postoperative BChE activity was lower or decreased in patients with POD compared to those without, we could not find any independent association of POD-development and the BChE activity. Furthermore, compared to other clinical screening tools that can indicate an onset of delirium such as CAM-ICU or NuDesc, our ROC-analysis of preoperative BChE activity showed only low sensitivity and moderate specificity for the predictive evaluation of the development of POD [23]. This makes our findings compatible with other studies involving patients with cardiothoracic surgery.

John et al. investigated 217 cardiosurgical patients who did not show significant differences in postoperative BChE activity between patients with and without POD, while preoperative activities or the decline have not been studied, respectively [24].

In the comprehensive CESARO-trial with 91 patients who received cardiac surgery neither pre- nor postoperative differences in BChE activity were able to stratify POD [7]. However, a different screening tool for POD-diagnosis was used (NuDesc), limiting profound comparisons to our investigation [23].

A sub study of the CESARO-trial by Michels et al. (2021) found significantly lower preoperative and postoperative BChE levels on three postoperative measurements plus a greater reduction in enzyme activity in patients with postoperative complications. However, their endpoint was a combination of POD, pneumonia, arrhythmia and acute renal failure [8].

A focused analysis showed no difference in perioperative decline of BChE activity between patients who developed POD and those who did not. Furthermore, the substudy only included patients with TAVI and no patients with cardiac surgery. Further inconclusive findings were provided by Adam et al., who reported a significant difference in BChE activity between POD- and non-POD-patients on the first postoperative day, but neither the following postoperative days nor preoperative data reproduced similar results [25].

The different study designs limit comparability, but in review of previous studies a high vulnerability of cardiac patients regarding the occurrence of POD underlines the need for further investigations. Incidence of POD in this study was almost 40%, which is slightly higher than reported incidences in other studies involving matching cohorts (Müller et al. 2019: 27.5%, John et al. 2017: 27.6%, Saha et al. 2020: 31.8%) [7, 9, 24].

In our study, POD-patients were older, had more premorbidities, less cognitive reserves and received longer procedures [9].

The neuroinflammatory hypothesis of POD suggests that a predominance of proinflammatory influences can lead to amplified systemic responses to further proinflammatory stimuli. The reason why older age represents – among other aspects – an independent risk factor for POD could be an imbalance of the innate and adaptive immune system in older patients, resulting in a low-grade proinflammatory state called ‘inflamm-ageing’ [26].

Contributing to this, patients with heart failure and myocardial infarction furthermore show increased levels of inflammatory cytokines [27, 28].

With regard to the interventions performed, commonly reported risk factors for the development of POD are the length of surgery and the use of cardiopulmonary bypass [29–31]. Tissue-trauma and hypo-/hyperperfusion during surgery leads to an activation of effector-cells of the immune-system, which can induce leucocyte migration, production of cytokines in mast cells or activation of macrophages and dendritic cells [32].

During the setup of the cardiopulmonary bypass the necessary cannulation, contact of blood with unphysiological surfaces and a higher concentration of bacterial endotoxins also represent a proinflammatory trigger [33].

We did not find a difference in POD incidence between patients with or without CPB (*p* = 0.489), which is in line with the results of the CORONARY-trial but stands in contrast to a study of O’Neal et al. from 2017, which found the CPB to be a major risk factor for the development of POD [34, 29].

The interaction of inflammatory processes in risk factors such as heart failure, coronary heart disease and older age in addition to the inflammatory stimulus of surgery and reduced cognitive reserves in older patients likely increases the vulnerability for developing POD [35].

BChE activity might still contribute to the development of POD, but we could not measure a significant and independent effect.

Instead, a substantial influence of age upon the preoperative BChE activity was observed. Every additional year of age decreased BChE activity by up to 21.9 U ml^−1^. In relation to the cholinergic anti-inflammatory pathway, this could possibly indicate a reduced anti-inflammatory reserve for pro-inflammatory stimuli such as surgery, if the reduction in cholinesterase-activity reflects overall acetylcholine availability. Considering the process of inflamm-ageing, the decline in preoperative enzyme-activity could also be a reflection of increased inflammatory processes in older patients. An age-related deficiency of its synthesis is also possible, since it is known for a wide-ranging group of other enzymes such as lactate dehydrogenase [36].

Postoperative BChE activity was, among other factors, particularly influenced by the type of surgery. Patients who received TAVI- or MitraClip procedure had higher postoperative BChE activity than patients who received bypass-, aortic- or valve-surgery. A reason for that could be a reduced release of proinflammatory cytokines and thus cholinergic anti-inflammatory response in less invasive procedures such as TAVI/MitraClip. The anticholinergic effect of several anesthetic drugs may have also influenced the BChE activity, but we decided against additional drug analysis in multivariable analyses, since they were indirectly represented by the type of the procedure with standardised anaesthesia protocols.

John et al. 2017 also proposed a possible influence of cardiopulmonary bypass and administration of blood transfusions on BChE activity [24]. Our findings partially support this hypothesis, since the use of CPB decreases postoperative BChE activity and the total number of administered RCCs negatively influences both postoperative BChE activity and its drop from pre- to postoperative. Since BChE is a plasma protein, the administration of RCCs and intravenous fluids in general could have had a dilutive effect, thereby decreasing the measured postoperative BChE activity. Whether or not an independent inflammatory response to exogenous blood products is possible remains difficult to decide, since patients on CPB also had longer surgery, received more RCCs in general and suffered more often from relative anemia, which also promotes inflammatory processes. The impact of anemia and the effect of blood transfusion on delirium have been investigated systematically before [37]. Blood transfusion for severe anemia might, on the one hand, trigger activation of endothelial cells and severe inflammatory responses upon blood product exposure.

On the other hand, a preceding severe anemia with a need for blood products leads to temporarily insufficient oxygen and glucose supply, subsequently resulting in cerebral injury [38].

Pre-existing factors such as age and chronic inflammation related to patients' morbidity are reflected by preoperative BChE activity. Postoperative BChE activity is additionally influenced by acute inflammatory reactions to triggers such as surgery. This is backed by our results regarding the inverse relationship of postoperative CRP-concentrations and BChE activities as well as findings by Zivkovic et al. [39]. Both acute and chronic inflammation can alter ACh metabolism and cause delirious symptoms, as proposed by the neuroinflammatory hypothesis of POD. However, it is not entirely clear why BChE activity decreases in proinflammatory environments. At first glance, it seems logical that reduced activity would increase the concentration of anti-inflammatory ACh and reduce inflammatory processes. However, both recent and past studies have consistently found lowered enzyme activities despite this apparent contradiction [7, 39–41].

This could mean that a downregulation of cholinesterase activity occurs when a higher ACh concentration is needed.

We conclude that BChE activity serves as an indirect marker of acute as well as chronic inflammation rather than a marker of a central cholinergic deficit. It should also be emphasised that the idea of measuring a central cholinergic deficit with a peripheral blood test is based on little data and should therefore carefully be reevaluated in further studies [42].

Our findings are underlined by the failure of interventional studies which assessed the effect of physostigmine application for prevention of POD [11]. Since inflammation can already be quantified by CRP- and procalcitonin-concentrations, the use of BChE activity as an additional marker is questionable. Recent studies also suggest that not a singular biomarker but rather a yet-to-be selected group of biomarkers representing different dimensions of organ dysfunction after surgery can increase specificity for delirium diagnosis [43].

In combination with a more diverse set of biomarkers, different methods of measuring the BChE activity such as mass spectrometry, could enhance both sensitivity and specificity and are worth considering. Screening for POD with established bedside instruments by trained and interdisciplinary teams continues to be the most important method and retains its relevancy pending further notice.

### Limitations

Strengths of this study are its prospective design and the extensive collection of intra- and postoperative data. Since pre- and postoperative data was collected, we were able to analyse the change in BChE activity. However, postoperative BChE activity was only measured routinely on day three and not on the first two postoperative days. Thus, early changes on day 1 or 2 might have been missed. Due to the nature of the POD syndrome with its interindividual dynamics with prompt but also delayed onset and the fact that inflammation does not diminish immediately after the intervention, the standardised determination of BChE at day 3 seemed reasonable to us.

Screening was performed until the third postoperative day only, which means that occurrence of POD thereafter would have been missed. Photometric measurement of enzyme activity can potentially be limited by the individual handling of samples, cross-reactions of agents, and interference of absorption with other substances found in the sample. However, it is a simple and cost-effective method that can be applied at the bedside. Measurements were done by the same person, so consistent handling of the samples was ensured.

## Conclusion

Preoperative BChE levels reflect the vulnerability of patients as indicated by age or history of stroke, while postoperative BChE levels reflect the subsequent inflammation of more invasive surgeries. Accordingly, they are also influenced by intraoperative RCC administration.

However, while these variables also play a large role for the development of POD, we could not find a direct link between BChE levels and POD. Therefore, the diagnostic relevance of the BChE as a marker for prediction or detection of POD in patients with cardiothoracic surgery or percutaneous valve replacement is questionable. More reliable tools to quantify inflammation already exist and thorough clinical diagnostics cannot be replaced in the detection of postoperative delirium.

### Supplementary Information

**Supplementary Material 1.** 

**Supplementary Material 2.** 

**Supplementary Material 3.** 

**Supplementary Material 4.**

## Data Availability

The data supporting the findings of this study are available from the corresponding author upon reasonable request.

## Abbreviations

BChE: Butyrylcholinesterase
3D-CAM: 3-Minute diagnostic interview for Confusion Assessment Method-defined delirium
CAM-ICU: Confusion Assessment Method—Intensive Care Unit
MoCA: Montreal-Cognitive-Assessment
POD: Postoperative delirium
AChE: Acetylcholinesterase
ACh: Acetylcholine
TAVI: Transcatheter aortic valve implantation
TNF-α: Tumor necrosis factor α
MST: Malnutrition Screening Tool
PHQ-2: (Score) patient health questionnaire
RCC: Red cell concentrate
FFP: Fresh frozen plasma
CBP: Cardiopulmonary bypass
ASA: (Score) American Society of Anaesthesiology
RASS: Richmond Agitation and Sedation Scale
CRP: C-reactive protein
ECRDW: Enterprise Clinical Research Data Warehouse
LDH: Lactate dehydrogenase
ROC: Receiver operating characteristic
CHF: Chronic heart failure
CHD: Coronary heart disease
MAP: Mean arterial pressure
BMI: Body-mass-index
NYHA: (Score) New York Heart Association
ECMO: Extracorporeal Membrane Oxygenation
HIV: Human Immunodeficiency Virus
OR: Odds ratio
NuDesc: Nursing Delirium Screening Scale
