# Supplementary material for: Butyrylcholinesterase activity in patients with postoperative delirium after cardiothoracic surgery or percutaneous valve replacement- an observational interdisciplinary cohort study
Source: BMC Neurol. 2024 Mar 1;24:80. doi: 10.1186/s12883-024-03580-9 (PMC10905803; doi:10.1186/s12883-024-03580-9)
Supplement: Supplementary file 1 — Supplementary Material 1. [file 12883_2024_3580_MOESM1_ESM.docx]

**Supplementary table 1: Association of pre-, intra- and postoperative parameters with decline in BChE-activity**

| **Preoperative parameters** | **n (patients) (%) / median (25^th^-75^th^ percentile)** | **Correlation (Rho)** | **p-value** |
| --- | --- | --- | --- |
| Age | 74 (64-79.5) | -0.136 | **0.037** |
| BMI (kg m^-2^) | 26.9 (24.1-30.1) | -0.090 | 0.168 |
| Female Sex | 89 (37.6%) |  | **0.040** |
| MoCA | 25 (22-26) | 0.014 | 0.832 |
| Haemoglobine (g dl^-1^) | 12.2 (11.0-13.4) | -0.014 | 0.833 |
| Alcohol abuse | 19 (8.0%) |  | 0.425 |
| Pulmonary embolism | 6 (2.5%) |  | 0.081 |
| History of stroke | 24 (10.1%) |  | 0.407 |
| Arterial hypertension | 51 (21.5%) |  | **0.045** |
| Type two diabetes | 65 (27.4%) |  | **0.014** |
| Adipositas | 62 (26.2%) |  | 0.171 |
| Myocardial infarction | 35 (14.8%) |  | 0.355 |
| Atrial fibrillation | 67 (28.3%) |  | 0.297 |
| Meningitis/encephalitis | 6 (2.5%) |  | 0.833 |
| Coronary heart disease | 206 (86.9%) |  | 0.115 |
| Heart failure | 130 (54.9%) |  | 0.179 |
| History of POD | 15 (6.3%) |  | 0.817 |
| Depression | 18 (7.6%) |  | 0.867 |
| Carcinoma | 29 (12.2%) |  | 0.252 |
| **Intra- and postoperative variables** | **n (patients) (%) / median (25^th^-75^th^ percentile)** | **Correlation (Rho)** | **p-value** |
| **Intraoperative variables** |  |  |  |
| Lactate (mmol l^-1^) | 1.5 (1.2-2.1) | 0.188 | **0.004** |
| Length of surgery (min) | 190  (147-234) | 0.357 | **<0.001** |
| MAP (mmHg) | 69 (66-74) | -0.202 | **0.002** |
| Total number of RCCs | 2 (0-4) | 0.308 | **<0.001** |
| Haemoglobine (g dl^-1^) | 8.9 (8.1-10.2) | -0.233 | **<0.001** |
| **Postoperative variables** |  |  |  |
| CRP (mg l^-1^) | 168 (103-230) | 0.316 | **<0.001** |
| Haemoglobine (g dl^-1^) | 10 (9.4-11) | -0.185 | **0.004** |
| Glucose (mmol l^-1^) | 5 (4.6-5.6) | -0.196 | **0.002** |
| Leukocytes (1000 µl^-1^) | 12.2  (9.9-16.1) | -0.199 | **0.002** |
| Potassium (mmol l^-1^) | 5.5 (5.1-6.0) | 0.314 | **<0.001** |
| Sodium (mmol l^-1^) | 143  (142-145) | 0.092 | 0.156 |
| Calcium (mmol l^-1^) | 2.1  (1.5-2.3) | 0.171 | **0.008** |
| Lactate (mmol l^-1^) | 2.2 (1.8-3.1) | 0.138 | **0.034** |
| Creatinine (µmol l^-1^) | 95 (81-116) | -0.003 | 0.967 |

Shown are total numbers of patients (% of total cohort) or median values (25^th^-75^th^ percentile). Univariate testing was done with *Mann-Whitney-U* test for categorial variables or *Spearman’s* correlation for continuous variables. 
Statistically significant results are shown in **bold**, p<.05 was considered significant. 
BChE, Butyrylcholinesterase; POD, Postoperative Delirium; MoCA, Montreal Cognitive Assessment; BMI, Body Mass Index; CPB, Cardiopulmonary bypass; CRP, C-reactive Protein RCC, Red cell concentrate.
