# Supplementary material for: Butyrylcholinesterase activity in patients with postoperative delirium after cardiothoracic surgery or percutaneous valve replacement- an observational interdisciplinary cohort study
Source: BMC Neurol. 2024 Mar 1;24:80. doi: 10.1186/s12883-024-03580-9 (PMC10905803; doi:10.1186/s12883-024-03580-9)
Supplement: Supplementary file 2 — Supplementary Material 2. [file 12883_2024_3580_MOESM2_ESM.docx]

**Supplementary table 2: Multivariable analysis of preoperative parameters and POD**

| **Variables** | **Odds-ratio** | **95% CI** | **Standard error** | **Regression coefficient** | **p-value** |
| --- | --- | --- | --- | --- | --- |
| Age | 1.027 | 0.998-1.057 | 0.015 | 0.027 | 0.065 |
| MoCA | 0.823 | 0.742-0.913 | 0.053 | -0.195 | **<0.001** |
| Type 2 DM | 1.508 | 0.800-2.842 | 0.323 | 0.411 | 0.204 |
| Coronary heart disease | 4.371 | 1.502-12.721 | 0.545 | 1.475 | **0.007** |
| Preoperative BChE-activity (U l^-1^) | 0.971 | 0.939-1.004 | 0.017 | -0.029 | 0.086 |

In multivariable analysis the independent association of preoperative parameters and POD has been tested using a binomial logistical regression model. p<.05 was considered significant. Statistically significant results are shown in **bold**,  POD, Postoperative Delirium; MoCA, Montreal Cognitive Assessment; DM, Diabetes mellitus; BChE, Butyrylcholinesterase.
