# Supplementary material for: Butyrylcholinesterase activity in patients with postoperative delirium after cardiothoracic surgery or percutaneous valve replacement- an observational interdisciplinary cohort study
Source: BMC Neurol. 2024 Mar 1;24:80. doi: 10.1186/s12883-024-03580-9 (PMC10905803; doi:10.1186/s12883-024-03580-9)
Supplement: Supplementary file 3 — Supplementary Material 3. [file 12883_2024_3580_MOESM3_ESM.docx]

**Supplementary table 3: Multivariable analysis of intra- and postoperative parameters and POD**

| **Variables** | **Odds-ratio** | **95% CI** | **Standard error** | **Regression coefficient** | **p-value** |
| --- | --- | --- | --- | --- | --- |
| Age | 1.058 | 1.020-1.098 | 0.019 | 0.057 | **0.002** |
| MoCA | 0.823 | 0.741-0.915 | 0.054 | -0.194 | **<0.001** |
| Type 2 DM | 1.631 | 0.831-3.199 | 0.344 | 0.489 | 0.155 |
| Coronary heart disease | 4.491 | 1.463-13.783 | 0.572 | 1.502 | **0.009** |
| Intraoperative RCCs | 2.033 | 1.005-4.113 | 0.359 | 0.710 | **0.048** |
| TAVI/MitraClip | 0.222 | 0.077-0.640 | 0.540 | -1.503 | **0.005** |
| Postoperative BChE-activity (U l^-1^) | 0.981 | 0.929-1.036 | 0.028 | -0.020 | 0.484 |

In multivariable analysis the independent association of intra- and postoperative parameters and POD has been tested using a binomial logistical regression model. Statistically significant results are shown in **bold**, p<.05 was considered significant. POD, Postoperative Delirium; MoCA, Montreal Cognitive Assessment; DM, Diabetes mellitus; RCC, red cell-concentrates; TAVI, Transcatheter aortic valve implanation; BChE, Butyrylcholinesterase.
