# Supplementary material for: Butyrylcholinesterase activity in patients with postoperative delirium after cardiothoracic surgery or percutaneous valve replacement- an observational interdisciplinary cohort study
Source: BMC Neurol. 2024 Mar 1;24:80. doi: 10.1186/s12883-024-03580-9 (PMC10905803; doi:10.1186/s12883-024-03580-9)
Supplement: Supplementary file 4 — Supplementary Material 4. [file 12883_2024_3580_MOESM4_ESM.docx]

**Supplementary table 4: Association of pre- and postoperative BChE-activity with pre-, intra- and postoperative parameters** 

| **Preoperative parameters** | **n (patients) (%)**  **/ median (25^th^-75^th^ percentile)** | **Correlation with preoperative BChE** | **p-value** | **Correlation with postoperative BChE** | **p-value** |
| --- | --- | --- | --- | --- | --- |
| Age (years) | 74 (64-79.5) | -0.193 | **0.003** | -0.004 | 0.957 |
| BMI (kg m^-^²) | 26.9 (24.1-30.1) | 0.062 | 0.340 | 0.177 | **0.006** |
| Female Sex | 89 (37.6%) |  | 0.249 |  | 0.157 |
| MoCA | 25 (22-26) | 0.041 | 0.531 | 0.092 | 0.158 |
| Hemoglobine (g dl^-1­­­^) | 12.2 (11.0-13.4) | -0.004 | 0.955 | 0.046 | 0.483 |
| Alcohol abuse | 19 (8.0%) |  | **0.028** |  | 0.222 |
| Pulmonary embolism | 6 (2.5%) |  | **0.011** |  | 0.559 |
| History of Stroke | 24 (10.1%) |  | **0.003** |  | **0.027** |
| Arterial hypertension | 51 (21.5%) |  | 0.187 |  | 0.414 |
| Type 2 diabetes | 65 (27.4%) |  | 0.535 |  | **0.040** |
| Adipositas | 62 (26.2%) |  | 0.619 |  | **0.018** |
| Myocardial infarction | 35 (14.8%) |  | 0.887 |  | 0.757 |
| Atrial fibrillation | 67 (28.3%) |  | 0.474 |  | 0.441 |
| Meningitis/encephalitis | 6 (2.5%) |  | 0.507 |  | 0.828 |
| Coronary heart disease | 206 (86.9%) |  | 0.451 |  | 0.598 |
| Heart failure | 130 (54.9%) |  | 0.787 |  | 0.241 |
| History of POD | 15 (6.3%) |  | 0.190 |  | 0.115 |
| Depression | 18 (7.6%) |  | 0.872 |  | 0.917 |
| Carcinoma | 29 (12.2%) |  | 0.557 |  | 0.347 |
| MoCA below 26 points | 153 (64.6%) |  | 0.264 |  | 0.330 |
| **Intra- and postoperative Parameters** |  |  |  | **Correlation with postoperative BChE** | **p-value (postoperative BChE)** |
| **Intraoperative Variables** |  |  |  |  |  |
| Haemoglobine (g dl^-1^) | 8.9 (8.1-10.2) |  |  | 0.346 | **<0.001** |
| Lactate (mmol l^-1^) | 1.5 (1.2-2.1) |  |  | -0.228 | **<0.001** |
| Length of surgery (min) | 190  (147-234) |  |  | -0.287 | **<0.001** |
| Mean arterial pressure | 69 (66-74) |  |  | 0.258 | **<0.001** |
| **Postoperative Variables** |  |  |  |  |  |
| CRP (mg l^-1^) | 168 (103-230) |  |  | -0.317 | **<0.001** |
| Lactate (mmol l^-1^) | 2.2 (1.8-3.1) |  |  | -0.185 | **0.004** |
| Number of RCCs | 2 (0-4) |  |  | -0.381 | **<0.001** |
| Haemoglobine (g dl^-1^) | 10 (9.4-11) |  |  | 0.288 | **<0.001** |
| Glucose (mmol l^-1^) | 5 (4.6-5.6) |  |  | 0.186 | **0.004** |
| Leukocytes (1000 µl^-1^) | 12.2  (9.9-16.1) |  |  | -0.188 | **0.004** |
| Potassium (mmol l^-1^) | 5.5 (5.1-6.0) |  |  | -0.279 | **<0.001** |
| Sodium (mmol l^-1^) | 143  (142-145) |  |  | -0.112 | 0.084 |
| Calcium (mmol l^-1^) | 2.1  (1.5-2.3) |  |  | -0.199 | **0.002** |
| Kreatinine (µmol l^-1^) | 95 (81-116) |  |  | -0.085 | 0.195 |

Shown are total numbers of patients (% of total cohort) or median values (25^th^-75^th^ percentile). Univariate testing was done with *Mann-Whitney-U* test for categorial variables or *Spearman’s* correlation for continuous variables. 
Statistically significant results are shown in **bold**, p<.05 was considered significant. 
BChE, Butyrylcholinesterase; POD, Postoperative Delirium; MoCA, Montreal Cognitive Assessment; BMI, Body Mass Index; CPB, Cardiopulmonary bypass; CRP, C-reactive Protein RCC, Red cell concentrate.
